# Vase-Life Monitoring System for Cut Flowers Using Deep Learning and Multiple Cameras

**DOI:** 10.3390/plants14071076

**Published:** 2025-04-01

**Authors:** Ji Yeong Ham, Yong-Tae Kim, Suong Tuyet Thi Ha, Byung-Chun In

**Affiliations:** Department of Smart Horticultural Science, Andong National University, Andong 36729, Republic of Korea; phy6134@anu.ac.kr (J.Y.H.); qkfkadpc@anu.ac.kr (Y.-T.K.); tuyetsuongha@gmail.com (S.T.T.H.)

**Keywords:** artificial intelligence, deep learning, microscope, monitoring, rose, vase life

## Abstract

Here, we developed a vase-life monitoring system (VMS) to automatically and accurately assess the post-harvest quality and vase life (VL) of cut roses. The VMS integrates camera imaging with the YOLOv8 (You Only Look Once version 8) deep learning algorithm to continuously monitor major physiological parameters including flower opening, fresh weight, water uptake, and gray mold disease incidence. Our results showed that the VMS can automatically measure the main physiological factors of cut roses by obtaining precise and consistent data. The values measured for physiology and disease by the VMS closely correlated with those measured by observation (OBS). Additionally, YOLOv8 achieved a high performance in the model by obtaining an object detection accuracy of 90%. Additionally, the mAP0.5 supported the high accuracy of the model in evaluating the VL of cut roses. Regression analysis revealed a strong correlation between the VL, VMS, and OBS. The VMS incorporating the microscope detected physiological and disease factors in the early stages of development. These results show that the plant monitoring system incorporating a microscope is highly effective for evaluating the post-harvest quality of cut roses. The early detection method using the VMS could also be applied to the flower breeding process, which requires rapid measurements of important characteristics of flower species, such as VL and disease resistance, to develop superior cultivars.

## 1. Introduction

The vase life (VL) and post-harvest quality of cut roses are crucial factors significantly influencing consumer purchase decisions and marketability [1,2]. Cut roses often face issues of early senescence due to ethylene impact, water stress, and disease infection during storage, transport, and sorting, resulting in a loss of marketability and reduced consumer satisfaction [3,4,5,6,7]. Water stress leads to petal wilting and discoloration, thereby reducing the freshness of cut flowers [3,8,9,10]. Among post-harvest diseases, gray mold disease (GMD) caused by *Botrytis cinerea* (BC) is the most significant necrotrophic pathogen negatively affecting the VL of cut roses [5,6,8,10,11]. Additionally, physical damage (e.g., flower wounding) during the distribution process can exacerbate quality deterioration [12].

The post-harvest VL assessment of cut flowers has traditionally relied on manual measurements, which are subject to human error because of visual inaccuracies, subjective judgments, and variability among evaluators [1,13,14,15,16,17,18]. Such inconsistencies can lead to significant differences in post-harvest quality evaluation, resulting in imprecise information for a quality grading system [12,16]. In cut flowers, quality and VL evaluations, such as of fresh weight and water uptake, are typically performed daily by investigators [19,20]. This approach is useful for monitoring overall physiological changes over time but requires considerable time and effort [21,22]. Furthermore, manual measurement often yields inconsistent values across evaluators and detects a specific factor only when the symptoms or disease spread visually [21,22]. Therefore, increasing demand exists for the development of rapid, accurate, and objective quality assessment systems to ensure consistency and reliability when evaluating cut flower quality.

Advancements in image analysis and artificial intelligence (AI) technology have enabled objective and efficient quality evaluations of various agricultural crops [1,17,18,19,23,24,25,26,27]. Multiple camera systems can effectively capture images from various angles, providing physiological and morphological data on plant quality [28,29]. Deep learning technology can automatically detect objects in image data, allowing for objective and consistent data classification [12,30,31]. The You Only Look Once (YOLO) algorithm offers high accuracy and efficiency in identifying regions of interest within complex image patterns, making it effective for real-time object detection tasks [30]. Camera systems combined with deep learning algorithms, such as YOLO, automatically analyze visual characteristics, such as color changes and disease occurrence, offering quantitative data analysis more objective and consistent than that by manual evaluations [12,16,30].

An automated assessment system based on AI and camera techniques can provide accurate and consistent quality information to consumers and thereby contribute to the expansion of cut flower consumption. Moreover, an accurate monitoring system can help breeders reduce time in developing a new variety that has a long vase life, which is an essential factor for cut flower cultivars. Thus, in this study, we aimed to develop a VL monitoring system (VMS) to automatically monitor physiological, morphological, and pathological factors based on image data obtained using multiple cameras and AI techniques. Additionally, we examined the predictive accuracy of the VMS in evaluating disease infection, water stress, and VL in cut roses.

## 2. Results

### 2.1. Changes in Vase-Life and Senescence Patterns According to Transport Methods

**Figure 1:**
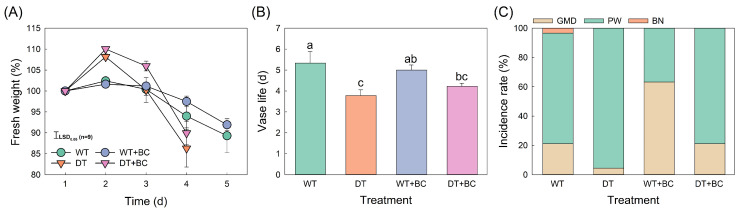
Effects of transport methods and *Botrytis cinerea* (BC) infection on the fresh weight (**A**), vase life (**B**), and incidence rate of senescence symptoms (**C**) in cut roses. Abbreviations: WT, wet transport; DT, dry transport; WT + BC, wet transport and BC inoculation; DT + BC, dry transport and BC inoculation; GMD, gray mold disease; PW, petal wilting; and BN, bent neck. Different letters (a–c) on vertical bars present significant differences among the treatments at *p* = 0.05 based on Duncan’s multiple-range tests. Vertical bars represent the standard errors (SEs) of the mean (*n* = 9).

The VL and senescence patterns differed significantly between dry (DT) and wet (WT) transports. The cut flowers in DT showed a large increase in fresh weight (FW) on day 2 and a rapid decline shortly thereafter (Figure 1A). Consequently, the VL of DT flowers was reduced by 2.1 d compared with that of the WT flowers (Figure 1B). Petal wilting (PW) was a major cause of VL in both transport methods (Figure 1C). After BC inoculation, the major cause of VL end was GMD (60%) in WT flowers whereas it was PW (80%) in DT flowers. This indicated that cut flowers were more susceptible to BC under WT conditions than that under DT conditions. 

### 2.2. Development of Prediction Models for VMS

**Figure 2:**
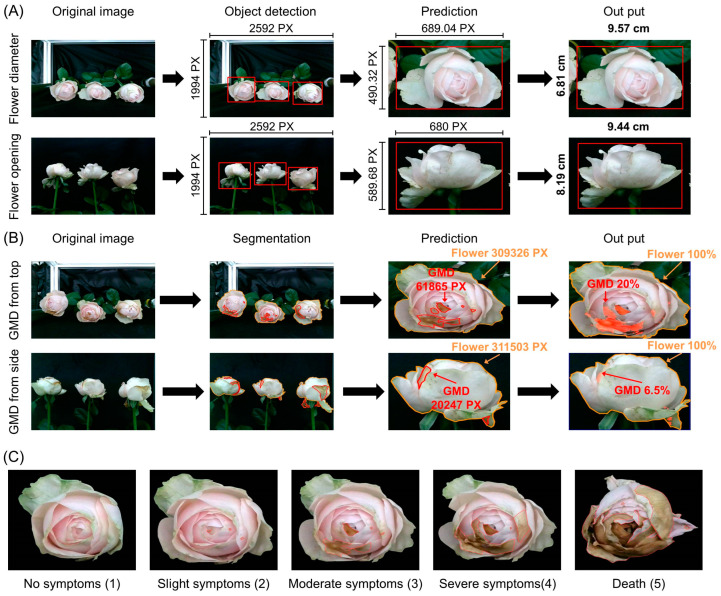
Prediction of flower diameter (FD) and flower opening (FO) using object detection (**A**), evaluation of gray mold disease (GMD) using segmentation in YOLOv8 (**B**), and the developmental index of GMD (**C**). The top and side indicate the images of flowers captured from the top and the side cameras, respectively. The object detection identified each flower head with a bounding box from the original image and measured the pixel (PX) of the image width, length, and height. The red bounding box indicates the designated region of the flower bud. The segmentation identified the region of GMD on the flower head with a polygon and PX of the area was measured. The PX of FD and FO was converted to cm and the PX of GMD was converted to percentages per total flower region by the prediction model. The GMD index in petals was evaluated based on the area of the infected petals according to an established scale: 1—no symptoms (0%); 2—slight symptoms (≤3%); 3—moderate symptoms (3–10%); 4—severe symptoms (11–50%); 5—death (>50%).

The object detection algorithm and segmentation of YOLOv8 were used for the VMS to measure the flower diameter (FD), flower opening (FO), and GMD levels of the cut roses. The flowers in the image were identified and labeled for the learning process, and the pixels (PX) (1 cm = 72 PX) in the bounding box of the flower head were measured to estimate the FD and FO (Figure 2A). The PX of the bounding box was converted to the size (cm) of the FD and FO based on the equations in the models, where R_p_, W_p_, L_p_, and H_p_ are the conversion value (72 pixels/cm), width of the flower image, length of the flower image, and bud image height (bounding box height PX), respectively.

(1) $$FD\;\left(cm\right)=\frac{L_{P}+W_{P}}{{2R}_{P}}$$

(2) $$FO\;(cm)=\frac{H_{P}\times W_{P}}{{2R}_{p}}$$

To predict GMD, the regions of GMD infection and flower heads were identified using a polygon labeling method (Figure 2B). The PX of the GMD region on the flower surface was converted to percentage per total flower region format based on the following equation:

(3) $$GMD\;area\;(\%)=\frac{PX\;of\;GMD}{PX\;of\;flower}\times 100$$

The water uptake (WU) of cut flowers was calculated as the difference between the initial and reduced PX of water on the vase images based on the following equation:

(4) $$Vase\;water\;\left(VW;\;g\right)={VW}_{i}\times \left(\frac{R_{w}}{R_{p}}\right)-{VW}_{n}\times (\frac{R_{w}}{R_{p}})$$

Here, VW_i_, VW_n_, *R_w_*, and *R_p_* are the vase water PX on the first day, vase water PX on the next day, reference weight (37.5 g cm^−1^), and conversion value (72 pixels/cm), respectively.

The fresh weight (FW) of the cut flowers was calculated by deducting the VW from the total vase weight using a digital balance.

Among the models employed for object detection, the YOLOv8 model demonstrated superior accuracy compared to the YOLOv5 models (Supplementary Table S1). Consequently, YOLOv8 was selected as the object detection method to measure flower quality in cut roses, focusing on GMD, FD, and FO. The performance of the YOLOv8 model constructed here was evaluated using precision and recall and mean precision (mAP). mAP effectively combines precision and recall into a single metric ranging from 0 to 1 and higher values indicate better model performance. The mAP0.5 is the mAP calculated when the predicted and actual object boundaries overlap by at least 50%. The mAP0.5–0.95 is the average mAP calculated over a range of IoU thresholds (from 50% to 95% in increments of 5%), providing a more rigorous assessment of detection performance. The overall model performance on the images from the top and side was high. mAP0.5–0.9 showed a slightly lower accuracy, although the mAP0.5 values on the top and side were 91.41 and 91.45, respectively (Table 1). This result indicates that the model had a high accuracy in predicting the physiological factors of cut flowers during the vase period.

### 2.3. Prediction of Flower Opening and GMD Infection Using VMS

**Figure 3:**
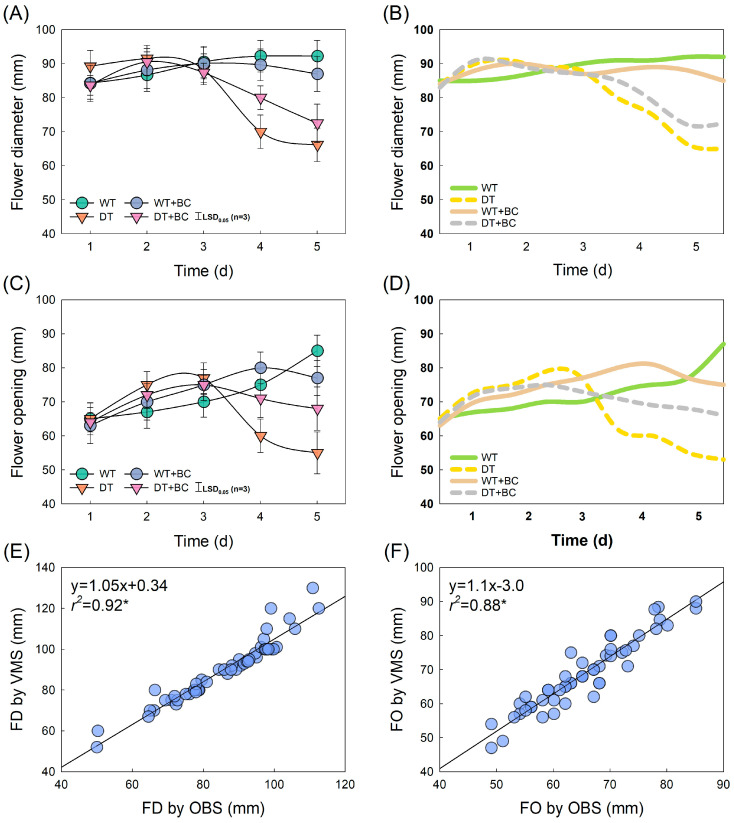
Comparison of changes in flower diameter (FD) and flower opening (FO) of cut roses between observed values (**A**,**C**) and VMS values (**B**,**D**). Simple linear regression analysis comparing FD-VMS and FD-OBS (**E**) and FO-VMS and FO-OBS (**F**). Abbreviations: VMS, vase-life monitoring system; OBS, observation; WT, wet transport; DT, dry transport; WT + BC, WT and *Botrytis cinerea* (BC) inoculation; DT + BC, dry transport and BC inoculation. Vertical bars represent the standard errors (SEs) of the mean (*n* = 3). Asterisk (*) represents a significant difference at *p* = 0.05 (*n* = 12).

The FD and FO values of WT flowers increased during the vase period whereas those of DT flowers rapidly decreased after day 3 (Figure 3A,C). The real-time changes in FD and FO by the VMS closely corresponded to those observed by observation (OBS) (Figure 3). BC inoculation (+BC) did not significantly affect the FD or FO of cut flowers. Simple linear regression analyses revealed that the FD and FO values measured by the VMS were closely correlated with those measured by OBS (*r*^2^ = 0.92, *p* = 0.05; and *r*^2^ = 0.88, *p* = 0.05) (Figure 3E,F). These results indicate that the VMS can accurately predict FO during the vase period in cut roses.

**Figure 4:**
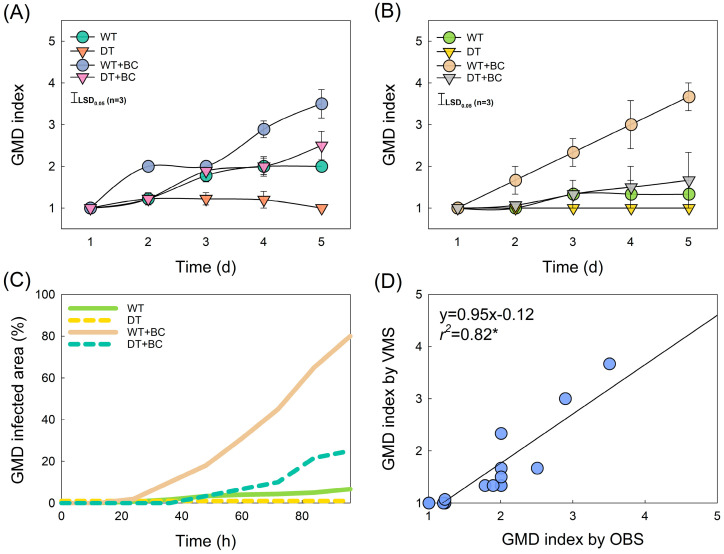
Effect of the treatments on gray mold disease (GMD) index and infected area (**A**–**C**) and simple linear regression analysis comparing observed (OBS) GMD and vase-life monitoring system (VMS) values (**D**). (**A**), OBS GMD index; (**B**), VMS GMD index; and (**C**), real-time GMD infected area. Abbreviations: WT, wet transport; DT, dry transport; WT + BC, wet transport and *Botrytis cinerea* (BC) inoculation; DT + BC, dry transport and BC inoculation. Vertical bars represent the standard errors (SEs) of the mean (*n* = 3). Asterisk (*) represents a significant difference at *p* = 0.05 (*n* = 12).

The GMD index was increased by +BC and further accelerated by WT conditions but decreased by DT conditions (Figure 4A). The GMD index and area measured by the VMS exhibited a pattern similar to that of the observations (Figure 4A–C). Correlation analysis comparing the GMD-index-VMS and GMD-index-OBS showed a high positive correlation (*r*^2^ = 0.82, *p* = 0.05) (Figure 4D). These results imply that the VMS can measure the FO and detect GMD in cut roses by obtaining precise and consistent data. These results revealed that real-time monitoring by the VMS can precisely predict the level of GMD development based on image data.

### 2.4. Evaluation of Fresh Weight and Water Uptake Changes Using VMS

**Figure 5:**
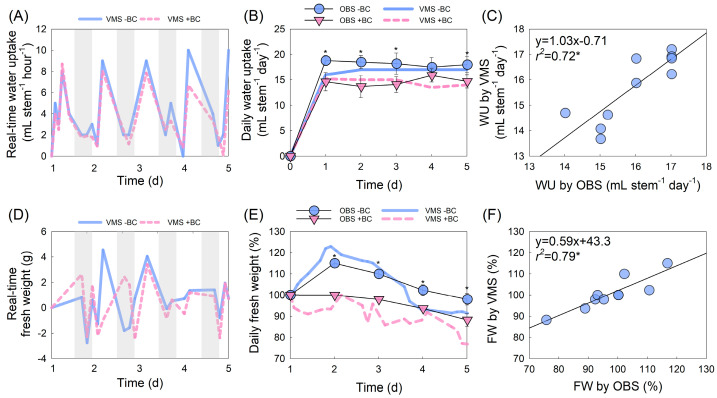
Changes in water uptake (WU) and fresh weight (FW) of cut roses. Real-time WU and FW (**A**), (**D**) were measured by a vase-life monitoring system (VMS). Daily WU and FW ((**B**), (**E**)) were measured by observation (OBS) and obtained by accumulation of VMS value. Simple linear regression analysis of WU (**C**) and FW (**F**) comparing VMS and OBS. Abbreviations: −BC, no *Botrytis cinerea* (BC) inoculation; +BC, BC inoculation. Vertical bars represent the standard errors (SEs) of the mean (*n* = 6). Asterisk (*) represents a significant difference at *p* = 0.05 (*n* = 12).

Real-time water uptake (WU) measured by the VMS showed diurnal fluctuations in both −BC and +BC flowers (Figure 5A). The WU significantly increased during the daytime, probably due to transpiration, and completely decreased at night. Cut flowers with +BC had a significantly decreased WU at a later stage. The daily WU of cut flowers decreased slightly with +BC, but no significant difference was observed between the VMS and OBS treatments (Figure 5B). A relatively high correlation (*r*^2^ = 0.72, *p* = 0.05) between WU-VMS and WU-OBS also supported the high accuracy of VMS measurements in the WU (Figure 5C).

Real-time FW showed diurnal fluctuations similar to those of WU in the early stage, although it showed an irregular pattern in +BC flowers (Figure 5D). The daily FW of cut flowers was significantly decreased by the +BC treatment (Figure 5E). The close correlation between FW-VMS and FW-OBS (*r*^2^ = 0.79, *p* = 0.05) also showed the high reliability of the VMS for FW measurement (Figure 5F).

### 2.5. Prediction of Flower Quality and Vase Life Using VMS

**Figure 6:**
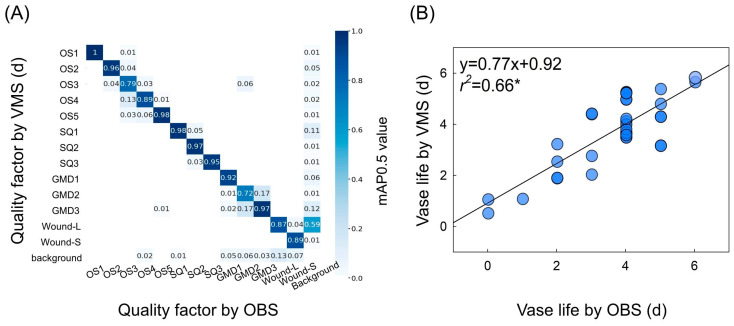
Accuracy of object detection on flower quality factors (**A**) and simple linear analysis comparing vase life (VL) predicted by the VMS and VL measured by observation (**B**). The object detection of YOLOv8 was performed on various quality factors based on mAP0.5. The quality factors include flower opening stage (OS) 1–5, stem quality (SQ) 1–3, GMD level (GMD) 1–3, petal wound large (Wound-L), and petal wound small (Wound-S). VL of cut flowers was predicted using YOLOv8 and ANN based on the object detection in (**A**). Abbreviations: VMS, vase-life monitoring system; OBS, observation. mAP is the evaluation index of detection accuracy. Asterisk (*) represents a significant difference at *p* = 0.05 (*n* = 12).

The performance of object detection based on mAP0.5 showed that the quality factors of cut roses were classified with good overall accuracy by the YOLOv8 model (Figure 6A). However, the object detection in opening stage 3 (OS3) and GMD3 (0.79 and 0.72) showed moderate accuracy (Figure 6A). This was probably due to a confusion during object detection and classification caused by overlapping visual features such as subtle color changes or petal shapes between the factors. The VL of the cut flowers was predicted using an artificial neural network (ANN) based on the object detection of the YOLOv8 model (Figure 6B). Although the model predicted the VL of cut roses somewhat accurately, a moderate relationship (*r*^2^ = 0.66, *p* = 0.05) between VL and OBS (Figure 6B) indicated that further improvement of the prediction model was necessary by enhancing object detection and increasing data quantity.

**Figure 7:**
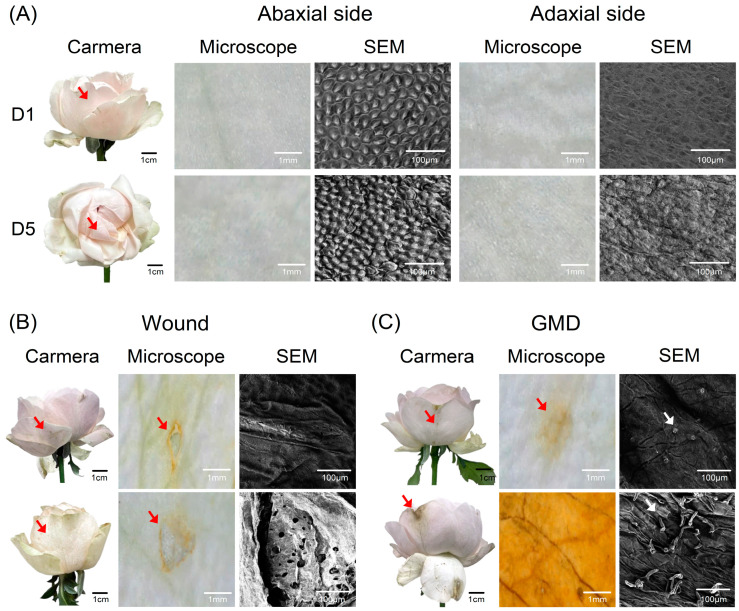
Images of cut rose flowers and petal surfaces on days 1 (D1) and 5 (D5) (**A**), symptoms of wounds on petal surfaces (**B**), and gray mold disease (GMD) on petals (**C**). Images of the petals of cut roses were captured from the petal abaxial (outer side) and adaxial (inner side) surfaces using cameras, microscopes, and scanning electron microscopy (SEM). The red arrows indicate the areas observed under the microscope and SEM. White arrows represent fungi associated with GMD.

It is generally difficult to identify and classify physiological and mechanical factors in the early stages based on camera images. The unclear object detection of the quality factors resulted in the reduced performance of the prediction model. To further enhance the accuracy of the model, portable microscope and scanning electron microscopy (SEM) methods were used to ensure that the senescence symptoms were detected by the VMS (Figure 7A–C). Changes in the turgidity of petal cells were detected in the images from the microscope and SEM (Figure 7A). SEM revealed cell disintegration on the petal abaxial and adaxial sides on day 5 (Figure 7A). The surfaces of the petals changed significantly because of turgor loss caused by the wilting of the entire flowers (Figure 7A). The changes in the petal surfaces observed in the microscope images were supported by the SEM images. Microscopy and SEM images also showed a clear distinction between flower wounds and GMD symptoms during the early stages (GMD index level 2) (Figure 7B,C). These results suggest that the addition of a microscope to the VMS can further increase object detection accuracy and subsequently enhance the performance of the prediction models in the VMS.

## 3. Discussion

The post-harvest quality and VL of cut roses are often negatively affected by water stress caused by DT and GMD infection [6,10,32,33,34]. In the current study, we found that DT and +BC conditions significantly reduced the VL and quality of cut roses. DT conditions lead to dehydration, particularly in cultivars susceptible to water stress, thereby reducing VL, despite their inhibitory effect on GMD development. These results are consistent with those of the previous findings, indicating that while GMD infection rates may decrease under dry conditions, dehydration can still negatively affect flower quality [5,19,32,33].

The application of the VMS and YOLOv8 object detection models represents a transformative step in post-harvest management. These techniques demonstrated high accuracy in assessing flower quality parameters and disease symptoms, offering a non-invasive alternative to traditional manual evaluations of cut flowers. Here, the VMS and YOLOv8 models were used to predict the quality and VL of cut flowers based on multicamera imaging. The use of the VMS allowed for the automatic collection of image data from various angles, significantly improving efficiency and facilitating the high-throughput data acquisition and extraction of diverse factors [35]. We evaluated the overall performance of the model based on the mAP, achieving a performance of 90% at an mAP0 of 0.5, indicating high prediction accuracy. Similar object detection performance was observed in other horticultural crops such as tomatoes, cucumbers, and eggplants [36,37,38]. The values measured by the VMS exhibited a strong correlation with those observed by the evaluators for the main quality factors in cut roses such as the FD, the FO, and GMD. The VMS was also capable of automatically measuring the FW and WU of cut flowers during the vase period. Real-time monitoring using the VMS revealed diurnal fluctuations in WU and subsequent FW, which were consistent with those of the previous findings [22,39]. These results provide a better understanding of the water relationships of cut flowers during the vase period, enabling the assessment of quality changes in response to environmental variables [21,22].

Here, the VL of cut roses was predicted using a VMS based on the opening stage, stem condition, GMD infection, and physical wounds. Although the predicted VL had a close correlation with that of the observations, detection errors were observed when attempting to identify early signs of visually detectable senescent symptoms due to the competitions among the factors and low image resolution. Subtle tissue changes such as those caused by early signs of GMD and wounds are challenging to distinguish through visual inspection and camera analysis [19,38]. Using SEM with high-resolution imaging and microscopy added further value by ensuring accuracy in symptom identification and distinguishing between overlapping factors, such as petal wounds and GMD symptoms, in the early stages [40,41]. SEM and microscopy also allowed the detection of subtle tissue changes in petals [42]. These technologies provided detailed insights into cellular-level changes, enhanced the precision of disease diagnosis, and improved the reliability of the VMS. However, the YOLOv8 model was developed using only one rose cultivar and the object detection at early stages in some factors showed relatively low accuracy due to completion among the factors. For a more accurate quality assessment and general applicability, further validation of the model with a larger dataset from various species and the additional integration of devices such as microscopes and spectrum cameras, along with enhancements in automation systems, is required for the VMS.

Flower sale for home use has been increased, especially through e-commerce markets, and consequently, ensuring a long VL based on a reliable evaluation system is important to promote flower sale. The reliance on manual selection and assessment systems can provide imprecise information for flower quality due to human errors and, in turn, lead to decreased consumer satisfaction and product value. The results demonstrated that the VMS developed in this study was remarkable in the accuracy of the post-harvest quality and VL prediction. An application of the VMS would not only improve the reliability on cut flower quality during marketing but also contribute to a reduction in the breeding period for new cultivars, ultimately expanding the flower industry.

## 4. Materials and Methods

### 4.1. Plant Materials

Cut roses ‘Unforgettable’ (*Rosa hybrida* L.) were purchased from Rosepia Agricultural Corporation (Jeonju, Republic of Korea) in May and July 2024. The cut roses were subjected to either the WT or DT method and transported to the Postharvest Physiology Laboratory at Andong National University, Republic of Korea, for 4 h. During transportation, only healthy rose flowers (non-latent infections and senescence symptoms) were selected for the experiments.

### 4.2. Botrytis Cinerea Growth and Fungal Suspension Preparation

BC (KACC40573) was obtained from the Korean Agricultural Culture Collection of the National Institute of Agricultural Sciences, Republic of Korea. Conidia were grown on potato dextrose agar (PDA, Difo Laboratories, Detroit, MI, USA) at 25 ± 1 °C for 2 weeks. A conidial suspension was prepared by adding 10 mL distilled water to the culture and shaking for 1 min. Thereafter, the suspension was gently filtered through four layers of sterile gauze, collected in a 100 mL Becher filter (Sangkong, Gyeonggi, Republic of Korea), and filtered twice to remove conidial clumps and mycelial fragments. The conidial suspension concentration was determined using a hemocytometer (Marienfeld, Bad Blankenburg, Germany) and adjusted to 10^5^ conidia mL^−1^ for inoculation.

### 4.3. Experiment 1: Real-Time Monitoring Changes in Physiology and Morphology

Cut roses were subjected to BC inoculation (+BC) by spraying 30 mL of a fungal suspension to induce GMD. The non-inoculated flowers (BC) were sprayed with the same volume of distilled water (DW). The cut flowers with +BC and −BC were stored in a refrigerator at 10 ± 1 °C, RH 50 ± 5%, under dark conditions for 24 h. Thereafter, the cut flower stems were trimmed to 45 cm with three upper leaves and placed in vases containing 400 mL DW. A cap with a hole (1 cm in diameter) was placed on the tops of the vases to maintain the cut stem upright position for consistent image capture. Cut flowers were placed in a controlled environment chamber (23 ± 1 °C; RH, 50–55%; 12 h photoperiod; light intensity of 20 µmol m^−2^ s^−1^) for VL assessment by evaluators.

### 4.4. Experiment 2: Measurement of VL and GMD Using VMS and Microscopy Cameras

The cut roses were wet-transported to the laboratory and subjected to either BC inoculation or DW spraying. The cut flowers with +BC and −BC were stored in a refrigerator for 24 h. Fungal inoculation and storage conditions were similar to those used in Experiment 1. Thereafter, the cut flowers were transferred to a controlled environment chamber (see Experiment 1) for VL and disease evaluation using a VMS.

### 4.5. Installation of VMS

**Figure 8:**
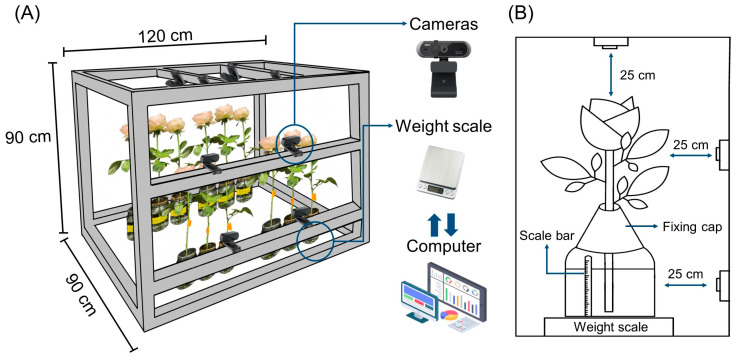
Structure of the vase-life monitoring system (VMS, (**A**)) and the diagram of cameras located at the up and side, cut flowers, and weight scale in the VMS (**B**). The image and weight data were transferred to a computer connected to the VMS to predict flower quality and conditions in real-time. The distance of cameras was 25 cm from the subject (cut flowers). A cap with a hole (1 cm in diameter) was used to maintain the cut stem upright position. A scale bar beside the vase was used as a reference in calculation of the pixel corresponding to the reduction in water level in the vase.

The VMS was manufactured using aluminum frames and consisted of 12 cameras and weight scales (Figure 8A). It was placed in a controlled environment chamber under 12 h day/night cycles (see Experiment 1). The distance between the cameras and the subject (cut flowers) was 25 cm (Figure 8B). Images of flowers were captured at one-minute intervals by cameras (APC930, ABKO, Seoul, Republic of Korea) located at the top and side frames (Figure 8A,B). At night, the light was turned on for 10 s per image. Each cut flower was placed on a weight scale to ensure precise monitoring of changes in FW and WU. The monitored images and weight values were transferred to a computer (intel^®^ Core ™ I7-14700KF CPU @ 3.4 GHz, Santa Clara, CA, USA) connected to the system. YOLOv8 and segmentation techniques were used to predict the quality and VL of cut flowers based on the image and weight data.

### 4.6. Assessment of Quality and Vase Life Using VMS

The images captured by the multiple cameras were analyzed using a deep learning algorithm to estimate the FD, FO, disease occurrence, FW, and WU of the cut roses. In Experiment 1, cut roses in each treatment were imaged using VMS from the top and side to monitor changes in physiology and disease progression. In Experiment 2, cut flowers were imaged using a VMS to verify the feasibility of automatic measurements of FW and WU.

### 4.7. Image Acquisition and Processing

**Figure 9:**
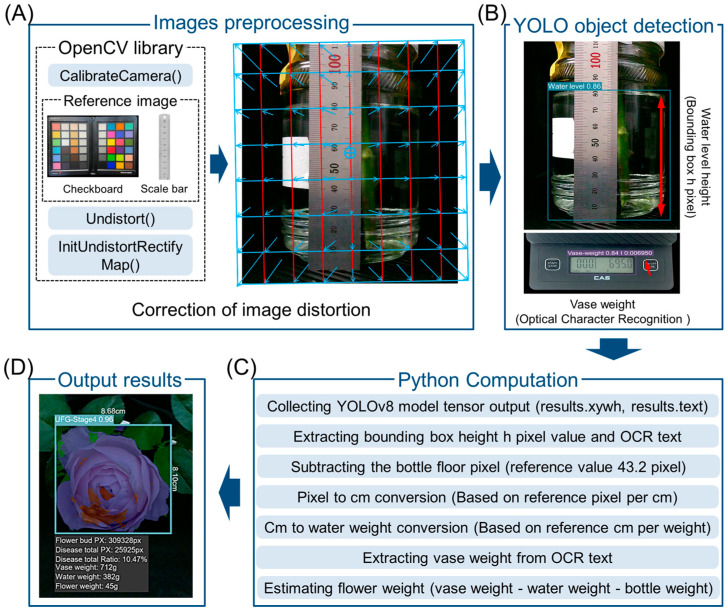
VMS image distortion correction and processing diagram of Python. (**A**) Image preprocessing using OpenCV library functions for distortion correction. (**B**) YOLOv8 object detection for extracting water level height (bounding box in pixels) and recognizing vase weight using Optical Character Recognition (OCR). (**C**) Python computation processes; YOLOv8 tensor output collection, bounding box height extraction, floor pixel subtraction, pixel-to-cm conversion, water weight estimation, OCR-based vase weight extraction, and flower weight estimation. (**D**) Output results displaying detected flower dimensions, disease area ratio, vase weight, water weight, and estimated flower weight. The blue arrows between red lines in (**A**) show an example of the distortion correction process, indicating how the original image is transformed into the corrected version. The Red arrow in (**B**) highlights the detected regions.

Reference image collection was performed every six hours using a reference board (reference ruler and a checkerboard) that was located beside the cut flowers. The reference images were used to correct for flower distance, tilt, and image adjustment values. Image distortion calibration was performed using the OpenCV library (version 4.10.0). The calibrateCamera() function was applied to the reference images to estimate the camera’s focal length and radial distortion coefficients. The images were then calibrated for distance and radial distortion using undistort() and initUndistortRectifyMap() functions (Figure 9A). YOLOv8 (Ultralytics, Frederick, MD, USA) was used to detect the water level in the vase and extract vase weight data. The bounding box height in pixels was measured for the water level and Optical Character Recognition (OCR) function was used to detect and extract the weight value displayed on the digital scale (Figure 9B). The collected YOLOv8 tensor output data was computed in Python version 3.10 (Python Software Foundation, Wilmington, DE, USA). The bottle floor pixel (reference value: 43.2 pixels) was subtracted to refine the detected water level. The extracted pixel values were then converted to centimeters based on a predefined reference pixel-to-cm. The water weight was estimated using a reference weight-per-cm. The extracted vase weight from OCR text was used to estimate the flower weight (Figure 9C). The final output result was computed in Python in real time. Flower diameter, disease area, vase weight, water weight, and estimated flower weight were displayed on the system interface for real-time monitoring (Figure 9D).

**Figure 10:**
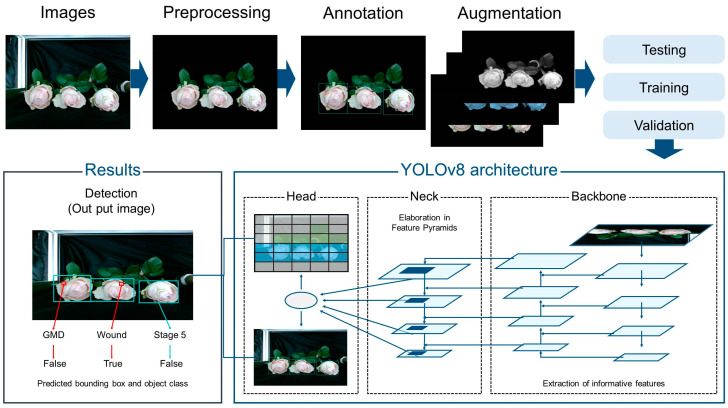
Structure of the YOLOv8 network model applied in this study. The flower image dataset underwent preprocessing, annotation, and augmentation to improve data quality and diversity. These processed images were then used to train the model, where the YOLOv8 architecture was divided into three key components: the backbone, the neck, and the head. The backbone was responsible for extracting essential features, which were then refined and structured by the neck, while the head made the final predictions regarding disease presence and estimated vase life. The performance of the model was evaluated through object detection metrics provided by the YOLOv8 system.

The flower image dataset was processed using YOLOv8 for quality factor detection, disease identification, and FD and FO measurements (Figure 10). In total, 50,000 images were collected and divided into 70%, 15%, and 15% (35,000, 7500, and 7500 images, respectively) for training, validation, and testing, respectively. Quality degradation factors were annotated with bounding boxes by an expert familiar with rose quality and disease symptoms using the Labellmg tool (MIT, Cambridge, MA, USA). YOLOv8 was implemented in Python version 3.10 and trained on a CUDA-enabled GeForce RTX 4060 GPU (NVIDIA Corporation, Santa Clara, CA, USA) for 200 epochs with a batch size of 16. Model performance was evaluated using precision, recall, and mAP, defined as follows:

(5) $$Precision=\frac{TP}{TP+FP}$$

(6) $$Recall=\frac{TP}{TP+FN}$$

(7) $$mAP=\frac{\sum_{i=1}^{k}{AP}_{i}}{k}$$

Here, *TP*, *FP*, *FN*, and *k* are true positive, false positive, false negative, and number of classes, respectively.

To identify an optimum image processing model, we evaluated the performance of Fast R-CNN, Single-Shot MultiBox Detector (SSD), and YOLOv5 in our previous experiments. The YOLOv5 model showed highest accuracy in predicting flower quality compared to other models [19]. Based on the result, we also compared the performance of YOLOv5 and YOLOv8 to identify most effective model in determining post-harvest quality of cut roses. The dataset (total of 582 images) used for the models included various senescence symptoms, disease levels, and developmental stages. The dataset was split into 70% for training, 15% for validation, and 15% for testing. All models were implemented using the PyTorch (version 2.0.1+cu118) open-source deep learning framework. The YOLO models were trained with a batch size of 16, and training went on for a total of 50 epochs.

**Figure 11:**
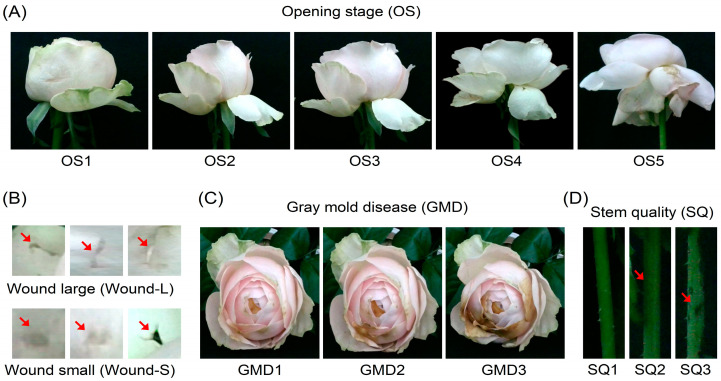
Annotation of flower opening stage (OS), petal wound, gray mold disease (GMD), and stem quality (SQ) in cut roses. (**A**) Five levels of flower opening stages (OS1–OS5); (**B**) petal wounds, large (Wound-L) and small (Wound-S); (**C**) three developmental stages of gray mold disease (GMD1–GMD3); and (**D**) three levels of stem quality (SQ1–SQ3). Red arrows indicate the wounded areas on the petals in (**B**) and surface damage affecting stem quality in (**D**).

An ANN model using the object values detected by YOLOv8 was used to predict the VL of cut flowers. We used a dataset of 200 cut roses, and the range of VL was 0–7 d. The VL of cut flowers was predicted based on cut flower quality indicators such as blooming stage, GMD index, petal wound, and stem condition (Figure 11).

To enable the quality assessment and VL prediction of cut roses using the prediction models, we labeled multiple factors related to flower quality (Figure 11). The OS ranged from tightly closed buds (OS1) to fully open blooms (OS5) (Figure 11A). Physical damage to the petals was classified as either large (Wound-L) or small (Wound-S) (Figure 11B). GMD development in the petals was labeled across three severity levels (GMD1, GMD2, and GMD3) (Figure 11C). Finally, stem quality (SQ) was categorized into three grades (SQ1, SQ2, and SQ3) based on stem conditions (physical damage or disease infection) (Figure 11D). These annotated images were used for object detection in the YOLOv8 model to predict the flower quality and post-harvest longevity of cut roses.

### 4.8. Microscope and SEM

Portable microscopy and SEM were used to observe and detect the early symptoms of GMD. Petal samples were collected on days 1, 3, 5, and 7. The petal surfaces were imaged at 125× magnification using a microscope (4 K 41MP; Eakins, Beijing, China) connected to a sensor (IMX334; SONY, Tokyo, Japan). After microscopic observation, the same areas of petals were cut into 5 × 5 mm sections, fixed in 2% osmium tetroxide solution (Electron Microscopy Sciences, Hatfield, PA, USA) in Petri dishes, and kept for 24 h in a hood. The petals were coated with Au ions for 60 s using an ion sputterer (Cressington Sputter Coater 108 Auto; Cressington Scientific Instruments, Watford, UK). Finally, the petal pieces were observed by SEM (VEGA II LMU; TESCAN, Brno, Czech Republic).

### 4.9. Evaluations of Flower Quality, Vase Life, and GMD Infection

Flower quality was measured daily at 10 a.m., including FD, FO, FW, and WU. The end of VL was considered when one or more senescence symptoms appeared: petal wilting (PW, ≥50% of petals losing turgor), petal discoloration (PD, ≥50% of petals turning blue), stem bending (stem bent at an angle exceeding 45°), or GMD (if more than 50% of petals were infected with BC) [43].

GMD index in petals was evaluated based on the area of the infected petals and the previous related scale [5,6]: 1—no symptoms (0%); 2—slight symptoms (≤3%); 3—moderate symptoms (3–10%); 4—severe symptoms (11–50%); 5—death (>50%).

### 4.10. Experiment Design and Data Analysis

All experiments were conducted twice. Thirty cut flowers were used in each treatment. Quality and VL were analyzed in blocks (three replicates with three flowers per replicate). The VMS analysis was conducted using three and six flowers per treatment in Experiments 1 and 2, respectively. All data were presented as means ± standard errors (SEs) and analyzed using SPSS version 22.0 (IBM, Somers, NY, USA). Significant differences were determined using a one-way analysis of variance (ANOVA), followed by Duncan’s multiple-range test at a significance level of *p* = 0.05.

## 5. Conclusions

Our results revealed that the VMS can automatically measure the main physiological factors, including GMD, FW, water status, and FO, by obtaining precise and consistent data. YOLOv8 achieved a high performance in the model by obtaining an object detection accuracy of 90%. The mAP also showed high accuracy across the VMS and the actual values in predicting flower quality. Additionally, the VL estimated by the VMS was similar to the observed VL. When the microscope was used as part of the VMS, the monitoring system could accurately detect factors in the early stages and ambiguous symptom development. These results indicate that the VMS combined with microscopy is an effective tool for the post-harvest quality assessment of cut roses and can be used in the breeding process to evaluate the quality and longevity of cut flowers.

## Figures and Tables

**Table 1:** Detection accuracy of prediction models based on the images from the side and top. The performance of the prediction models by YOLOv8 was evaluated by precision, recall, and mAP. Precision is the percentage of true positives (correctly detected objects) out of all the detected objects; recall is the percentage of true positives out of all the existing objects in the dataset; mAP is the evaluation index of the detection accuracy.

Image	Precision (%)	Recall (%)	mAP0.5 (%)	mAP0.5–0.9 (%)
Top	90.85	88.53	91.41	74.01
Side	86.46	91.94	91.45	70.60

## Data Availability

Data will be made available on request.

## Supplementary Materials

The following supporting information can be downloaded at: https://www.mdpi.com/article/10.3390/plants14071076/s1, Supplementary Table S1: Comparison of detection accuracy between YOLOv5 and YOLOv8 models.
