# Supplementary material for: Vase-Life Monitoring System for Cut Flowers Using Deep Learning and Multiple Cameras
Source: Plants (Basel). 2025 Apr 1;14(7):1076. doi: 10.3390/plants14071076 (PMC11991080; doi:10.3390/plants14071076)
Supplement: Supplementary file 1 [file plants-14-01076-s001.zip › plants-3470342-supplementary.pdf]

Supplementary Tables

Table S1. Comparison of detection accuracy between YOLOv5 and YOLOv8 models. The performance of the prediction models was evaluated by Precision, Recall, and mAP. Precision is the percentage of true positives (correctly detected objects) out of all the detected objects; Recall is the percentage of true positives out of all the existing objects in the dataset; mAP is the evaluation index of the detection accuracy.

Models	Precision (%)	Recall (%)	mAP0.5 (%)	mAP 0.5-0.9 (%)
YOLOv8x	89.95	81.50	82.04	68.48
YOLOv5x	84.12	76.08	77.17	62.12
YOLOv8s	78.55	73.12	75.40	61.47
YOLOv5s	71.64	75.12	71.76	59.20
